# Comparative Study of Corrosion Performance of LVOF-Sprayed Ni-Based Composite Coatings Produced Using Standard and Reducing Flame Spray Stoichiometry

**DOI:** 10.3390/ma17020458

**Published:** 2024-01-18

**Authors:** Abdelhek Idir, Francesco Delloro, Rassim Younes, Mohand Amokrane Bradai, Abdelhamid Sadeddine, Gabriela Marginean

**Affiliations:** 1 Laboratory of Mechanics, Materials and Energetic (L2ME), Faculty of Technology, University of Bejaia, Bejaia 06000, Algeriamdamokrane.bradai@univ-bejaia.dz (M.A.B.); abdelhamid.sadeddine@univ-bejaia.dz (A.S.); 2 MINES ParisTech, PSL Research University, MAT—Centre des Matériaux, CNRS UMR 7633, BP 87, 91003 Evry, France; francesco.delloro@minesparis.psl.eu; 3 Institute for Mechanical Engineering, Westphalian University of Applied Sciences Gelsenkirchen Bocholt Recklinghausen, Neidenburger Str. 43, 45897 Gelsenkirchen, Germany

**Keywords:** thermal spray, flame stoichiometry, microstructure, porosity, corrosion performance

## Abstract

Coating efficiency and quality can be significantly improved by carefully optimizing the coating parameters. Particularly in the flame spray method, the oxygen/fuel ratio, which is classified as oxidizing flame stoichiometry (excess oxygen) and reduces flame stoichiometry (excess acetylene), and spray distance are the most critical factors, as they correlate significantly with coating porosity and corrosion performance. Hence, understanding the effects of these parameters is essential to further minimize the porosity, improving the corrosion performance of thermally sprayed coatings. In this work, a NiWCrBSi alloy coating was deposited via the oxyacetylene flame spray/Flexicord-wire (FS/FC) method. The effect of the flame oxygen/fuel ratio and spray distance on the microstructure properties and corrosion behavior of the coatings was investigated. Afterwards, the microstructure, phases’ compositions, spray distance, and corrosion performance were studied. The equivalent circuit model was proposed, and the corrosion mechanism was discussed. The obtained results highlight that the oxygen-to-fuel ratio is a promising solution for the further application of flame spray/Flexicord-wire (FS/FC) cermet coatings in hostile environments. Depending on the flame’s oxygen/fuel ratio, careful selection of the flame stoichiometry provides low porosity and high corrosion performance.

## 1. Introduction

Nowadays, mild steel is widely used due to its exceptional weldability, ductility, and low-cost machinability. It is utilized in many applications, including signs, fences, and the automotive and construction industries. These components have to meet increasingly stringent requirements in terms of the corrosion resistance properties of metallic materials, so that they can meet design requirements for long service life and high reliability [1,2]. At present, surface modification and coating technology is emerging as a cost-effective alternative for protecting engineering components against wear and corrosion damage [2]. Among various coating methods, thermal spraying is an advanced materials processing technique that involves heating or melting particles and depositing them onto the surface to produce thick coatings that improve the surface properties of materials [3,4]. Furthermore, one of the most common and cost-effective solutions to overcome the corrosion damage of industrial components is to apply a protective coating onto the component’s surface [2,3]. The protective coating could be prepared via various thermal spray techniques, including a High-Velocity Oxygen Fuel (HVOF) spray, arc thermal metal spray (ARC), atmospheric plasma spray (APS), and Low-Velocity Oxygen Fuel spray (LVOF) technologies.

Depending on the thermal energy source, thermal spraying can be divided into electric arc spraying, plasma spraying, and flame/combustion spraying, where the feedstock-materials used are in powder, wire, or rod form [2,5]. For the HVOF spray, thermal energy is produced by fuel combustion (oxygen/gas), while for the plasma spray technique, a plasma plume is used as a high enthalpy source, which helps in the sufficient melting of feedstock powder. Due to the supersonic speed of the flame, the kinetic energy of the droplets is higher in the HVOF spray than in the plasma spray [6,7]. One of the biggest disadvantages of the APS spray is the fact that the radially injected feedstock into the plasma plume undergoes insufficient melting and has insufficient speed due to shorter dwell time of the droplets in the heat source. Despite the availability of powder feedstock-materials, the powder spray technology is restricted to powders ranging from 10 to 100 μm. However, due to their low moment of inertia, small particles are unable to follow gas flow trajectories, resulting in poor fluidity and nozzle clogging, and, thus, the cooled particles were deposited onto the surface of the substrate [7,8]. According to the literature, coating quality depends on various factors, such as the size of the sprayed particles, the deposition angle and speed, the spray distance, the substrate temperature, the feed speed, and, of course, the deposition technique [4,9,10]. Consequently, all of these factors should be carefully controlled to provide the optimal properties for each application. NiCrBSi alloy coatings are also commonly prepared by using powder-spray techniques, including HVOF [10,11], APS [2,5], ARC [12,13], and LVOF [14,15]. In particular, previous research has been devoted exclusively to the potential interest of nickel-based alloys, and a great deal of effort has gone into meeting the challenge of providing new high-strength materials for specific protection in hostile environments. Based on its chemical composition, the NiCrBSi alloy provides excellent wear and corrosion resistance. It has been reported that the addition of Si and B increases the self-fluxing properties of nickel alloys [4,10], while chromium is essential for corrosion resistance. Boron is commonly added to nickel alloys to depress the melting point, while carbon enables the formation of carbides [16,17]. The microstructural properties of thermally sprayed NiCrBSi were studied by [18,19,20]. Many researchers have focused on the effect of WC, Ti, and TiB_2_ reinforcements on the wear behavior of thermally sprayed NiCrBSi coatings [21,22,23]. The effect of the addition of Mo and Ta on the microstructural properties and high-temperature wear resistance of the thermally sprayed NiCrBSi coating was studied by [24,25]. The obtained results show that the coating exhibits high microstructure stability and wear resistance. The dissolution of nickel-based alloying elements in corrosive environments is also highly suitable for the further production of corrosion product films, acting as a barrier to electrolyte diffusion and promoting high corrosion resistance [26,27]. According to the literature [28,29], the oxidation of the elements Cr, Mo, and W leads to the formation of a passivation film that prevents the diffusion of the electrolyte towards the substrate. Researchers in surface engineering and chemistry have highlighted corrosion propagation associated with micro-structural defects as a major challenge. Souza et al. [30] identified the main critical issues related to the corrosion performance of thermal spray coatings.

In general, understanding the corrosion performance of HVOF-sprayed coatings is complex because of their particular multiphase microstructure comprising the formation of new and nonequilibrium phases. According to the literature [30], in cermet coatings, carbide phases dissolve strongly in the metal matrix, which deeply affects their corrosion mechanisms. Further studies have demonstrated that carbide particles may induce galvanic corrosion in the matrix, while crevice corrosion may occur at the carbide–matrix interface [31]. In particular, it is widely believed that particle fusion is a fundamental factor for good coating quality. Using thermal spraying technology, e.g., atmospheric plasma spraying (APS), can produce an ultra-high melting point because of the high plasma temperatures (8000–14,000 K) and particle speeds in the range of (100–300 m/s) [32]. As indicated in the literature, the atmospheric plasma spraying (APS) technique uses the plume as a heat source and deposits the droplets onto substrates, using a layer-by-layer method. This method results in a typical microstructure, which is associated with non-bonded boundaries. Chang-jiu et al. [33] reported that, in plasma spray coatings, non-bonded boundaries result from restricted diffusion between lamellae or particles. Furthermore, according to the literature [34], non-bonded boundaries in as-sprayed NiCrBSi coatings provide diffusion paths for the electrolyte in a corrosive environment.

Despite the lower particle temperature (2300 K to 2700 K) and particle velocities below 100 m/s achieved in the flame spraying process, significant advantages in terms of the optimization of the processing spray parameters could be attained. Therefore, further attention was devoted to the corrosion performance of thermally sprayed coating using the LVOF spray technique. Nevertheless, in the LVOF spray method, a fundamental parameter is thus represented by the acetylene-to-oxygen equivalence ratio. As a result, the flame temperature depends on the oxygen-to-fuel ratio, which may control the coating quality. Optimizing flame spray parameters is therefore of fundamental importance if high corrosion coating resistance has to be achieved. In practice, better optimization of flame stoichiometries could increase flame temperatures up to 34,000 k [32]. None of the published articles examined the relationship between the microstructure, flame stoichiometry, and spray distance on the corrosion performance of thermally flame spray/Flexicord (FS/FC) coating, based on the literature. The main goal of this study was to deliberately focus on the effect of the oxygen/fuel ratio, i.e., oxidizing flame stoichiometry (excess oxygen) and reducing flame stoichiometry (excess acetylene), and the spray distance on the corrosion performance of flame spray/Flexicord-wire (FS/FC) NiWCrBSi coating. The electrochemical behavior of the NiWCrBSi coating was investigated in artificial seawater solution. The coating was characterized via scanning electron microscope (SEM) and energy dispersive spectroscopy (EDS), the corrosion mechanisms were investigated by using SEM, and then the electrical equivalent model was proposed. The results obtained provide detailed insight into the suitability of the optimized spray parameters for corrosion applications in marine environments of LVOF (FS/FC) NiWCrBSi coating.

## 2. Materials and Methods

### 2.1. Coating Preparation

**Figure 1:**
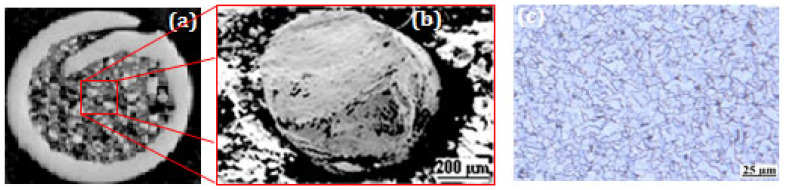
(**a**,**b**) SEM image of Flexicord RocDur62 and (**c**) optical image of the substrate.

**Figure 2:**
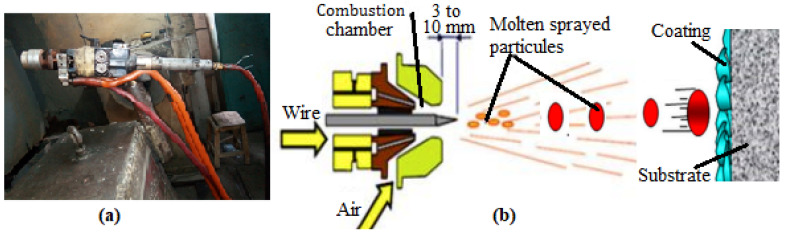
Schematic of flame spraying process, (**a**) flame spray gun, and (**b**) formation of droplets.

Ni-based super alloy (NiWCrBSi), commercially available on Flexicord Rocdur62 (Saint-Gobain, Courbevoie, France) (see Figure 1), was used as a feedstock material. The chemical composition, as defined by the manufacturer, is given in Table 1. The LVOF coating was carried out using a Master Jet 2 (Saint-Gobain, France) gun, as shown in Figure 2. Detailed spray parameters are presented in Table 2.

The substrates were machined from mild steel bars (E335) in the form of cylindrical pins 10 mm in diameter and 15 mm in length and then used to deposit the coating. Before the spraying process, the surface of the samples was shot-blasted using an abrasive medium. The nominal chemical composition of the steel substrate was as follows: C <0.1%; Mo <0.007%; Mn <0.231%; P <0.050%; N <0.03%; Cu <0.119%; Si <0.044%; V <0.01%; S <0.010% and Fe balance.

### 2.2. Coating Characterization

After coating deposition, the samples were cut into cross-sections and polished with 180–4000 SiC paper. Finer polishing was then carried out using 7, 3, and 1 µm diamond pastes. Finally, the samples were cleaned with acetone and dried with compressed air. The microstructure analysis was investigated via SEM, using an FEI NanoNova SEM 450 (Hillsboro, OR, USA) scanning electron microscope equipped with an energy-dispersive X-ray microanalysis (EDS) system. The porosity analysis was carried out by analyzing at least 5 SEM images at 200 magnifications, using the ImageJ software (version 1.53h, Rasband, W.S. ImageJ, U.S. National Institutes of Health, Bethesda, MD, USA).

### 2.3. Potentiodynamic Polarization Procedure

The corrosion behavior of the samples was assessed experimentally via the potentiodynamic polarization technique, using a potentiostat/galvanostat (VoltaLab PGZ 301) electrochemical workstation, controlled by a VoltaMaster 4 (version 4.0.21289). equipped with a conventional three-electrode cell. A saturated calomel electrode (SCE) was used as the reference electrode, a platinum electrode was used as the counter electrode, and the surface of the samples was used as the working electrode. All the electrochemical measurements were examined in 3.5 wt. %NaCl aqueous solution at room temperature (~22 ± 0.5) °C. Prior to corrosion tests, the sample was embedded in epoxy fixed to a copper wire, leaving an exposed surface area of 10 mm × 10 mm. Before each experiment, all the surfaces of the samples were polished and given a mirror finish in a water suspension, using SiC abrasive papers (grade 400 to 2000). Potentiodynamic polarization measurements were taken from −1000 mV to +1000 mV, with a scanning rate of 0.5 mV/s.

### 2.4. Electrochemical Impedance Spectroscopy (EIS) Procedure

The Electrochemical Impedance Spectroscopy (EIS) measurements were evaluated from 12 h up to 96 h of immersion times in 3.5 wt. % NaCl solution. The frequency range was extended from 100 kHz to 10 MHz. The corresponding mean values were used in the relative diagrams. The equivalent electrical circuit (EEC) was determined using the ZsimpWin software (E-chem Software, version 3.21, Ann Arbor, MI, USA), and the results were fitted to the proposed equivalent circuit (EC) model. The quality of the fitting results was expressed in terms of the chi-squared value (χ^2^). Each experiment was repeated three times to prove the reproducibility of the results.

## 3. Results and Discussion

### 3.1. Microstructure Characterization

**Figure 3:**
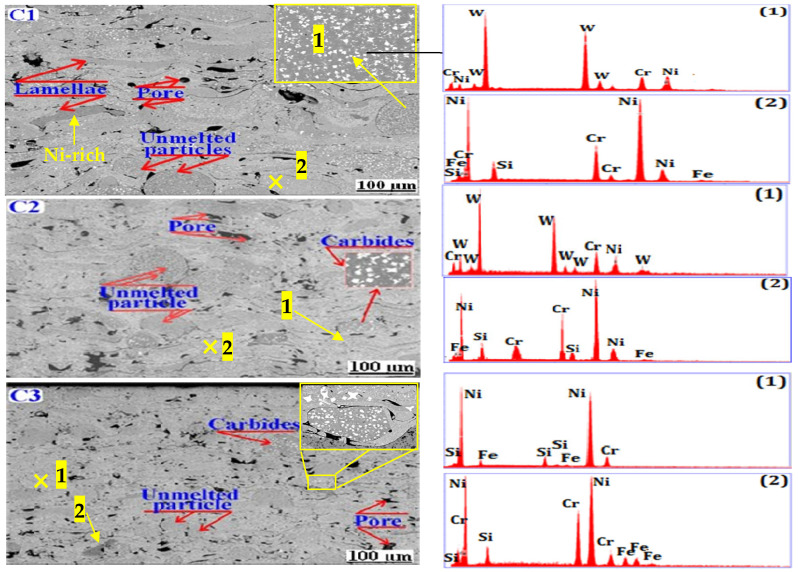
SEM micrographs of the as-sprayed coatings in cross-section.

Figure 3 shows the microstructure of the as-sprayed coatings C1, C2, and C3. The corresponding EDS analysis was carried out. The coatings’ thickness was as follows: 1.1 mm, 1.3 mm, and 1 mm for the samples C1, C2, and C3, respectively.

The cross-section micrographs display a typical lamellar structure with elongated splats of molten powder, pores, and carbide phases (white areas) randomly distributed throughout the coatings. The SEM microstructures showed that there was no decarburization of the WC carbide phases and no formation of brittle W_2_C and W_3_C phases. In turn, the absence of microcracks showed that the processing parameters resulted in producing high-quality coatings.

**Figure 4:**
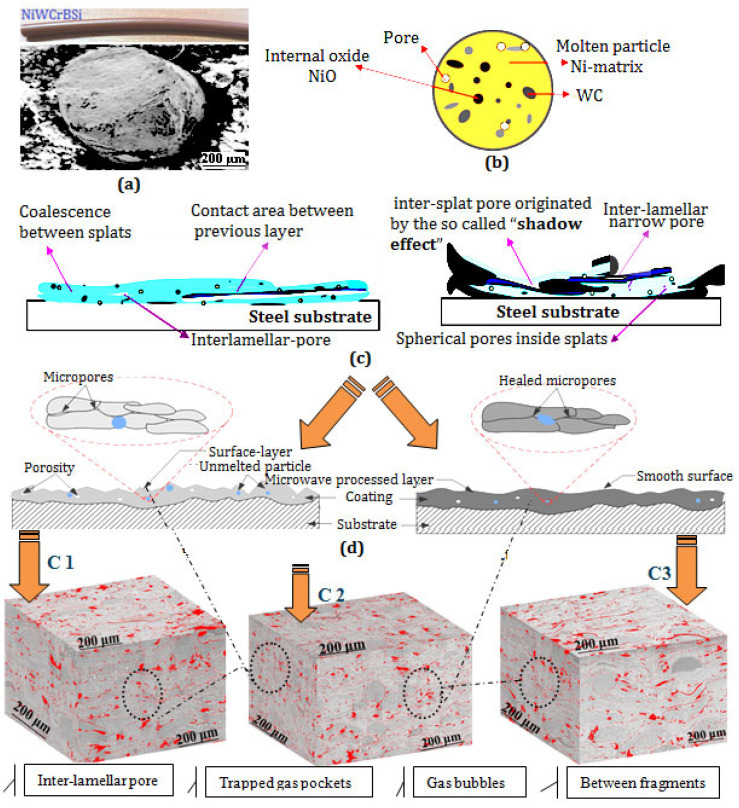
Schematic view of the porosity formation mechanism: (**a**) Flexicord Wire RocDure62, (**b**) particle, (**c**) formation of porosity in the coating, and (**d**) porosity of the cross-section of the coating.

An EDS analysis was carried out for each grayscale region associated with each phase of the coating. The analyzed regions and the corresponding EDS spectra were marked with the numbers 1 and 2, respectively. The EDS analysis revealed the presence of the chemical elements Ni, Cr, Si, Fe, and W, while elements B and C are too lightweight to be detected by EDS. Similar results were reported by Kazameret et al. [3]. Moreover, boron acts as a deoxidizing element, and, during spraying, it is also preferentially oxidized, and the B_2_O_3_ formed is quickly and completely eliminated via evaporation; therefore, no oxides were observed in the coatings. By carefully examining the microstructure of the coatings, the cross-sectional micrographs display that different submicron pores are randomly distributed throughout the coatings. Each pore type arises from different mechanisms and is represented schematically in Figure 4. The observed structures areas, i.e., black areas, may originate from the shrinkage of the molten particles, the inter-lamellar boundary or incomplete inter-splat connections, and the cooling rates of the sprayed particles during flame spraying. Furthermore, as the spray distance increases, the porosity level decreases, and the structures become denser (see Figure 4). The porosity level of the sprayed coatings was calculated by analyzing five SEM image micrographs at 200 magnifications, using ImageJ software (version 1.53h). The obtained results revealed that coatings C2 and C3 showed a porosity level of 8.461 ± 1.2% and 7.138 ± 7.3%, respectively, while coating C1 showed higher values of 9.678 ± 1.1%.

### 3.2. Corrosion Behavior of As-Sprayed Coatings

**Figure 5:**
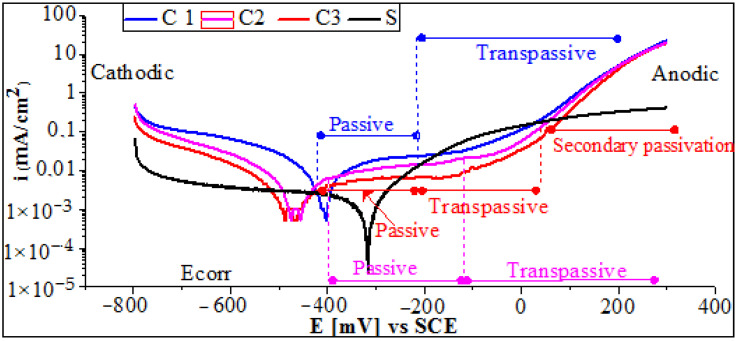
Potentiodynamic polarization curves of the LVOF sprayed coatings tested in 3.5% NaCl solution.

As mentioned previously, the as-sprayed coating has a large thickness and a rough, wavy surface after deposition, which is also a result of the different flame spray parameter used. Prior to electrochemical tests, the samples were ground and polished. Accordingly, the coating thickness ranged from 870.9 ± 4 μm to 876.6 ± 2 μm, and the surface roughness of the samples was Ra <0.2 μm. The corresponding potentiodynamic polarization curves (Tafel plots) were recorded after 60 min in NaCl solution and are shown in Figure 5. The obtained electrochemical parameters are listed in Table 3.

It can be seen that the polarization curves can be divided into four distinct regions, namely cathodic, anodic, passive, and transpassive regions. It is evident from Figure 5 that, when the potential is anodic, no active current peaks are observed, indicating that the coatings are passivated immediately. For coating C3, the passive region is found to extend over a relatively wide range of potentials, from around −400 VSCE to −20 VSCE. In the passive region, the current density is practically stable over a wide range of potentials. Furthermore, it can be seen that the secondary transformation of the transpassive layer is indicated exclusively by slight oscillations in the current between −110 VSCE and 80 VSCE. Therefore, the breakdown of the protective film begins at about 80 VSCE, and secondary passivation occurs between 80 VSCE and 400 VSCE. However, the corrosion potentials exhibit a difference of 80 mV. Nevertheless, these differences are relatively small for coatings C2 and C3, exhibiting a very similar corrosion resistance. It is also noted that the steel substrate suffers from serious corrosion by losing electrons when compared to the tested coatings. The cathodic branch was larger than that of the coating, indicating the severe corrosion damage of the steel. The determined experimental values displayed in Table 3 show that the corrosion potential of the steel substrate shifts to a more positive value as compared to the coatings. Further values from Table 3 confirm that the steel is more vulnerable to corrosion than coatings C1, C2, and C3. The Icorr of the coated and uncoated samples was 84.66 ± 3.1, 5.844± 0.9, 3.865± 0.7, and 2.027±0.3 for the steel substrate coatings C1, C2, and C3, respectively.

In fact, when the uncoated steel substrate is immersed in NaCl solution, the C^l−^ in the corrosive medium directly attacks the surface of the substrate, and the dissolution of Fe occurs, so that further selective desolation of the Fe results in sever corrosion damage. However, the corrosion process is hindered when using a coating. It is also believed that due to the Ni-rich matrix of the coating, the W atomic radius difference is not large, resulting in the formation of Ni-W components. The gap between the lamellae boundary and the pores is larger, creating the corrosion medium in the Cl^−^ preferential adsorption at these defects, causing coating surface damage. However, the rapid dissolution of Ni in the pore provides Ni^2+^, which further attracts Cl^−^ to maintain electro-neutrality. In addition, when the Ni-W area comes into contact with the corrosive medium, Ni may preferentially dissolve, and it reacts very easily with H+ to produce Ni^2+^, resulting in the formation of a NiO/Ni(OH)_2_ protective film [35]. Other cations, such as, Cr^3+^, Si^2+^, and Fe^2+^, can be provided from the dissolution of the NiCrBSi alloy elements and converted to the corresponding oxides thereafter. According to the literature [28,29], chromium still has a significant role in material composition and corrosion resistance. Therefore, a protective chromium oxide acting as a passive film is encouraged. SEM micrographs of the coating after 96 h in a NaCl solution as shown in Section 4 below, shows that, in the cross-section, the dissolution of the coating element leads to the formation of corrosion products, which hinder the diffusion of the electrolyte to reach the surface of the substrate. This phenomenon is strongly encouraged when using a higher oxygen/acetylene flame ratio (excess acetylene), resulting in high corrosion resistance. Depending on the oxygen/fuel flame ratio, the lowest current density was obtained with an excessive acetylene ratio, while the highest current density was obtained with an excessive oxygen ratio.

### 3.3. Electrochemical Impedance Spectroscopy (EIS) Behavior

**Figure 6:**
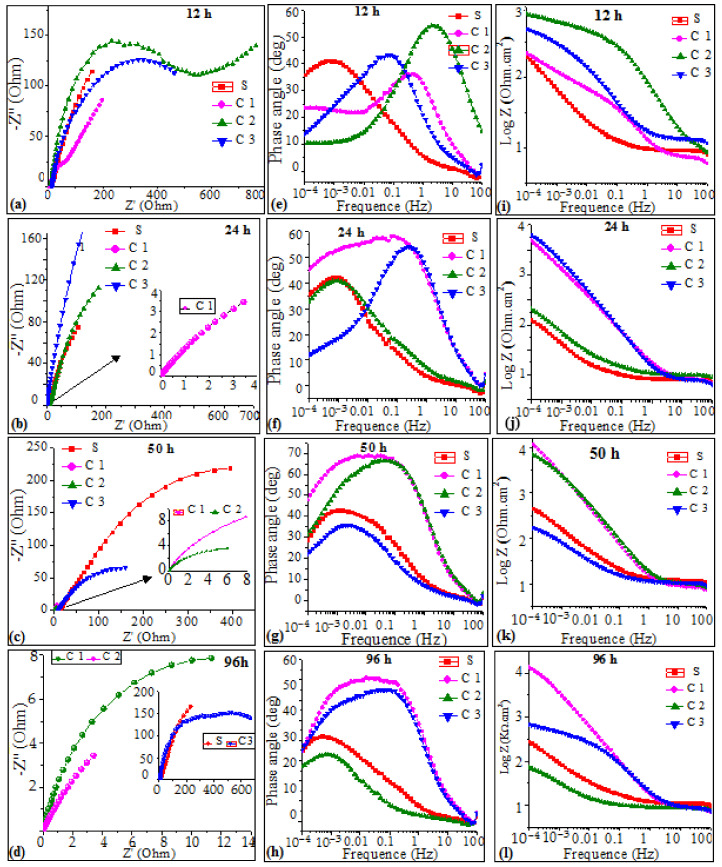
EIS data for coatings at different exposure time in artificial seawater: (**a**–**d**) Nyquist diagram, (**e**–**h**) Bode diagram, and (**i**–**l**) phase diagram.

Figure 6 shows the Nyquist, Bode, and impedance–frequency plots obtained from 12 h up to 96 h of immersion in artificial seawater at 25 °C. Nyquist plots from 12 to 96 h of immersion (Figure 6) reveal an imperfect semicircle over the entire frequency range, typical of chemical reactions where Na^+^ and Cl^−^ ion diffusion is relatively limited in the initial stage. The corresponding Bode plots exhibit the presence of one or two time constants because of the correspondingly wide frequency range of each peak. However, for coatings C1 and C2, at a 12 h immersion time, two capacitive loops are clearly visible on the Nyquist plot (see Figure 6). Consequently, there are two inflection points on the Bode diagram (Figure 6), indicating that coating C2 exhibits two-time constants.

**Figure 7:**
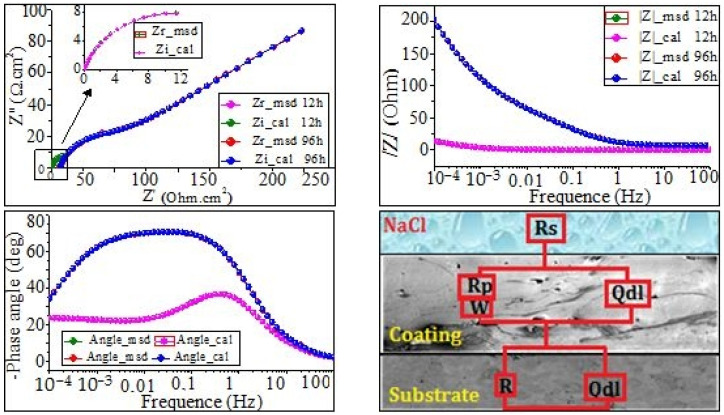
Electrical equivalent circuit and the corresponding EIS fitting results of coating C1 after 12 and 96 h in NaCl solution.

The low-frequency loop reflects the corrosion process of the coating, while the high-frequency loop is likely to be associated with the coating defects, such as unmelted particles, inter-lamellar boundaries, and porosities. According to the literature, the presence of two maximum time constants in the Bode diagrams (Figure 6) could be related to the presence of a carbide phase in the coating, as evidenced by microstructural features, such as WC grains, that provide additional interfaces to the surface of the coated samples. The impedance is also lower at higher frequencies for all samples (see Figure 6). This may suggest that the diffusion of ions does not contribute to the impedance at higher frequencies. The complexity of the microstructure properties of thermally sprayed Ni-based alloy coatings could lead to the suggestion that the corrosion process of these alloys may exhibit instability during immersion times and result in low-frequency (LF) dispersion, with evidence of inductive behavior caused by the development of pitting [5,10]. Furthermore, pitting formed, and corrosion products precipitated on the surface of the working electrode. This pitting phenomenon was studied in depth and was highlighted by SEM cross-section images after corrosion. As discussed previously in Section 1, the coating exhibits defects. The depressed semicircle in the Nyquist diagrams obtained in the present work is due to submicron defects, frequency dispersion, surface inhomogeneity, and the presence of a nonuniform passive film. Similar results were reported in the literature [33,34]. It is observed that these semi-circles are followed by a tail, as Warburg impedance (W), which was well defined and much pronounced as a diffusion element in the low-frequency region. As seen from the Nyquist plots in Figure 7, a straight line with a slope that was much closer to 45° appears at low frequencies. 

**Figure 8:**
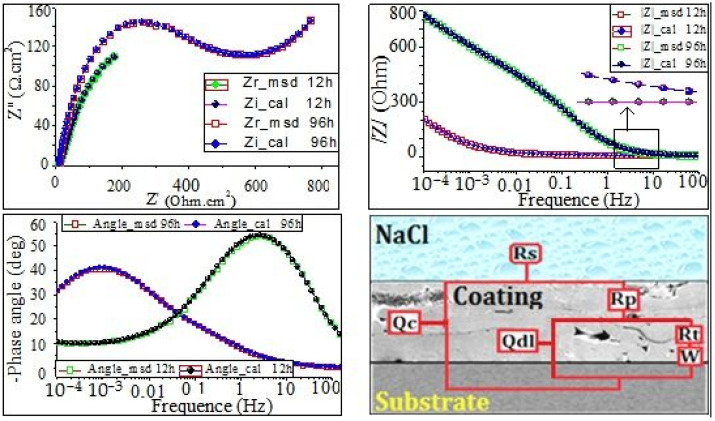
Electrical equivalent circuit and the corresponding EIS fitting results of coating C2 after 12 and 96 h in NaCl solution.

**Figure 9:**
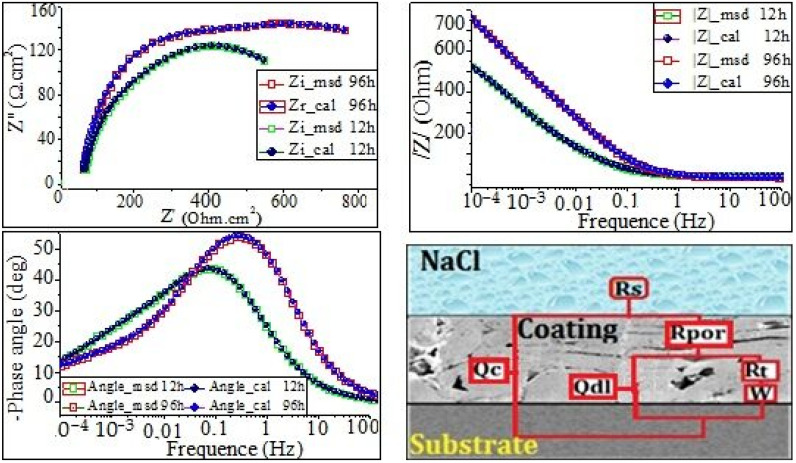
Electrical equivalent circuit and the corresponding EIS fitting results of coating C3 after 12 and 96 h in NaCl solution.

The equivalent circuits were determined by using the ZSimpWin program (Figure 7, Figure 8 and Figure 9), and the fitting parameters are summarized in Table 4 and Table 5. The equivalent circuit of coating C1 was carefully proposed and presented in Figure 7. It consisted of Rs(Qdl(RpW))(QdlRt) and was used to fit the results obtained after 12 h and 96 h of immersion in NaCl. Meanwhile, the proposed equivalent circuit of coatings C2 and C3 after 12 h and 96 h immersion times in NaCl is Rs(Qc(Rp(Qdl(RtW)))). The proposed model in Figure 8 and Figure 9 consist of the following:

Rs is the resistance of electrolyte solution; (ZCPE)$Q_{c}$ is the impedance of the constant phase element (CPE)$Q_{c}$, which characterizes the capacitance of oxides layers and corrosion products; R_por_ is the resistance of the pores and pits; Rt is the resistance of the film; and (ZCPE)$Q_{dl}$ is the impedance of the constant phase element (CPE)$Q_{dl}$, which characterizes the electrical double layers.

The electrical circuit was modeled by the Warburg impedance (W) in series with R film. 

According to the literature [27], the admittance of the W element can be expressed as follows:

(1) $$Y-W\left(ɷ\right)-Y_{0}\times {\left(Jɷ\right)}^{1/2}$$

(2) $$Y_{0}=l_{e}/(R_{D0}\times \sqrt{D})$$

where ɷ is angular frequency, $R_{D0}$ is the diffusion resistance, and D is the diffusion coefficient.

Because of the presence of inter-lamellar boundaries, unmelted particles, and pores, bulk capacitive behavior can be expected. Since pure capacitance is difficult to achieve, in the present study, the constant phase element (CPE) was often used to simulate capacitance. In the ZsimpWin program, the CPE impedance is also defined as follows:

(3) $$Z_{CPE}=\frac{1}{{Q\left(J\omega \right)}^{n}}$$

According to the literature [33,34,35], the exponent n is a usual parameter that is very useful to demonstrate the nature of the impedance (Z_CPE_); it is obtained from the slope of (log (|Z|) vs. log (f)) plot. Note that 0 ≤n≤ 1, whereas, when n=−1, the Q value corresponds to an ideal inductor. The following expressions may be derived through a dimensional analysis:

(4) $$n=0\;\to Z_{CPE}=\frac{1}{Q}=R$$

(5) $$n=0.5\;\to Z_{CPE}=\frac{1}{{Q\left(J\omega \right)}^{n0.5}}=W$$

(6) $$n=1\to Z_{CPE}=\frac{1}{QJ\omega }=C$$

**Figure 10:**
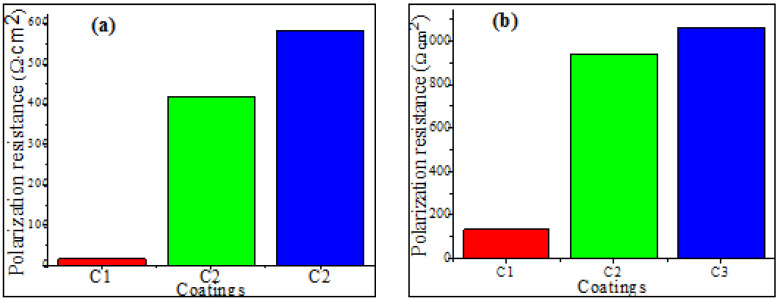
Evolution the of polarization resistance as a function of the immersion time in 3.5 wt. % NaCl solution (**a**) after 12 h and (**b**) after 96 h.

From Figure 7, Figure 8 and Figure 9, it can be clearly seen that the simulation results are in excellent agreement with the experimental data. The EIS-simulated results in Table 4 and Table 5 have practically a 90% confidence interval. The chi-squared (χ^2^) values range from 9.41 × 10^−4^ to 9.99 × 10^−4^ for the coating C1, and 4.9 × 10^−3^ to 7.97 × 10^−4^ for the coating C2 and C3, respectively, demonstrating excellent agreement between the experimental and simulated data. Since the polarization resistance ($R_{p}$) values can provide a correlation with the corrosion resistance occurring in microdefects (i.e., micropores), they were also used to express the pitting corrosion rate. It can be seen that both the $R_{por}$ and $R_{t}$ were quite lower, whereas ($Q_{c}$) and ($Q_{dl}$) were quite higher during the earlier stage of immersion, indicating that the corrosion process of the coating occurred. However, by extending the corrosion time, the $R_{t}$ increased rapidly, corresponding to the rapid decline in the ($Q_{c}$). This is because of the existence of micropores in the coating (Figure 8 and Figure 9); the corrosive solution could penetrate into the coating through the micropores. Furthermore, the $R_{por}$ at 96 h of immersion time for coating C2 is larger than that for coating C3, while the $R_{t}$ of coating C2 is obviously smaller than that of coating C3. The above results indicate that both $R_{por}$ and $R_{t}$ were the main resistances of the corrosion process; the polarization resistance, $R_{p}(R_{p}=R_{por}+R_{t}),$ is used to determine the corrosion resistance of the coating. Figure 10 shows that the polarization resistance, $R_{p}$ (i.e., $R_{por}$ + $R_{t}$), is lower in the first stage of immersion time. However, this value becomes larger at higher immersion times. On the other hand, from Figure 10a, it can be seen that the $R_{p}$ values are about 15.79, 419.44, and 581 Ωcm^−2^ for the coatings C1, C2, and C3 for the short immersion times (12 h). Meanwhile, the polarization resistance, $R_{p}$, increased drastically to 133.514, 941.5, and 1063.8 Ω cm^−2^, respectively, for long immersion times 96 h (see Figure 10b). These results could lead to claims that, the larger the $R_{p}$ value, the greater the pitting corrosion resistance, (i.e., the lower the corrosion rate). Conversely, the lower the $R_{p}$ values, the higher the pitting corrosion rate.

### 3.4. The Corrosion Mechanisms

**Figure 11:**
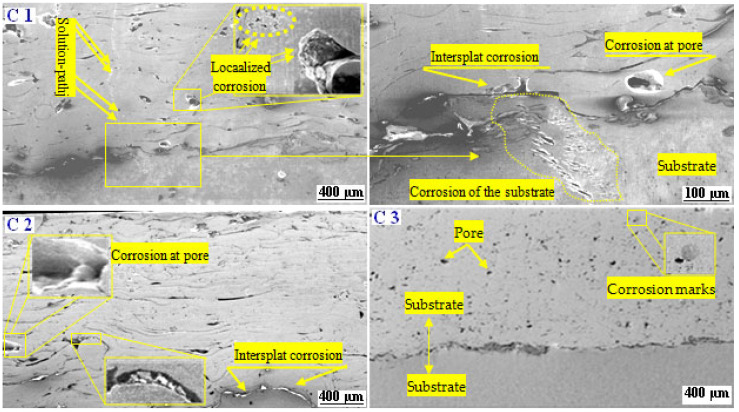
SEM cross-section morphology after 96 h immersion in simulated seawater.

The corrosion mechanisms of the as-sprayed coatings after a 96 h immersion in NaCl solution were studied in cross-sections via SEM (Figure 11). The image analysis revealed that the corrosion marks of coating C1 were very prominent, with many shallow and deep corrosion holes (see the dotted line circle at higher magnification in Figure 11C1). Also, on the corroded cross-section, we observed that coating C1 clearly displayed some big corroded pits, which were of various sizes, up to 20 μm. In addition, many pits are evidenced which may be caused by the corrosion spalling of splats, indicating localized corrosion (see Figure 11C1). The SEM cross-section image at high magnification on the right side of Figure 11C1 showed that the coating was severely damaged, with pathways in which the electrolyte reached the substrate in a certain region, as shown by the dashed line in Figure 11C1. As expected, the attacked regions are confined essentially at the coating’s microdefects. According to the literature [10,30], the microgalvanic and/or micro-crevice corrosion mechanism can occur via microdefects. Pathways allowing the electrolyte to reach the substrate were observed through the thickness of the coating. This feature is most likely the result of electrolyte diffusion through the defects, which could lead to accelerate the corrosion process resulting from the potential difference between the surface and the bottom of the pores. The cross-section examination of both coatings C2 and C3 after 96 h of immersion (Figure 11C2,C3) showed that, unlike the C1 coating, no pathways allowing the electrolyte to reach the substrate was observed. In contrast, the micrograph coating C2 displays that the pores became relatively larger because the original micropores were damaged. Typically, the galvanic corrosion of lamellar boundaries can lead to large corrosion paths, thus forming voids. Consequently, we can clearly confirm that a part of the corrosion mechanism is generated by grain boundaries, as well as inter-splat boundaries, as shown on the right side of Figure 11C3. Over the course of time, the interaction between the electrolytic solution and the dissolution of the metallic phases of the coating leads to the formation of a large quantity of corrosion products. As a result, the self-filling of the coating porosity attributed to the dissolution of the metallic phases of the coating and the passive oxide film will take place. As a result, the pores are filled with corrosion products that act as a barrier to the diffusion of the electrolytic solution.

**Figure 12:**
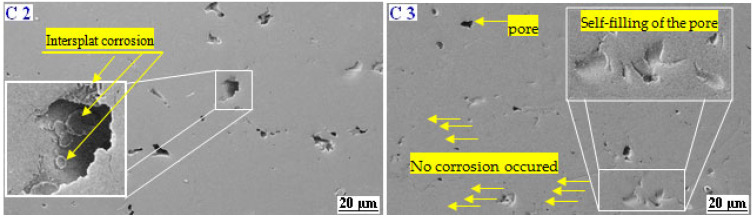
SEM details of the morphology of the coatings C2 and C3 after 96 h in NaCl solution.

Detailed SEM micrographs at a higher magnification revealed that some spheroidal corrosion products had formed in the coating porosities, as shown by the double arrow in Figure 12C3. As discussed earlier in the in Tafel polarization results (Figure 12C3), the coating C3 exhibits a larger transpassive domain and secondary transpassive domain, indicating ideal repassivation behavior. The EIS results provide further evidence that electrolyte access into the coating thickness is hindered and that the corrosion damage occurred only on the top surface of the coating. Consequently, it is also indicated that the top of coating C3 presented higher roughness than the coatings C1 and C2. There is therefore no interfacial corrosion or corrosion inside the coating. Figure 12C3 shows only small marks of the corrosion that appeared in the pores of the coating. As discussed further below, the corrosive medium concentrates in the pores, which were represented by the Warburg diffusion coefficient in the fitted EIS equivalent circuit.

## 4. Conclusions

A strategy to deposit a NiWCrBSi coating with a crack-free microstructure via a conventional frame spraying was presented. By using flame spray/Flexicord-wire (FS/FC) with different oxygen-to-fuel ratios and generating highly hot metallic droplets with modified flame stoichiometry, we enabled the creation of an essentially oxide- and crack-free, high-performance coating to be achieved. This microstructure displays outstanding corrosion resistance in artificial seawater. Depending on the oxygen/fuel flame ratio, the lowest current density was obtained with an excess acetylene ratio, while the highest current density was obtained with an excess oxygen ratio.

The potentiodynamic polarization results showed that the corrosion mechanism of NiWCrBSi coatings could originate from a cyclic process of passivation, penetration, and repassivation. An excess acetylene flame ratio with increasing spray distance results in a larger transpassive domain and a secondary transpassive domain, indicating ideal repassivation behavior. 

The SEM morphology, after a long immersion time, showed that the reducing flame-sprayed coating with a longer spray distance was not prone to pitting or any form of local damage when exposed to the NaCl environment; this could justify the fact that the self-filling of the coating porosity, attributed to the dissolution of the metallic phases of the coating and the passive oxide film, strongly occurred. As a result, the diffusion of the electrolyte was hindered.

These results highlight the fact that the oxygen-to-fuel ratio is a promising solution for the further application of flame spray/Flexicord-wire (FS/FC) cermet coatings in harsh, corrosive environments. Furthermore, according to the corrosion results, the abovementioned strategy is promising to be applied in other alloys, such as Inconel 718, Inconel 625, and other nickel-bases super-alloys.

## Figures and Tables

**Table 1:** Chemical compositions of the Flexicord (Rocdur62) wire.

Elements	Ni	W	Cr	B	Si	Fe	C
wt.%	Bal	9.5	14.3	2.8	3.7	3.5	0.6

**Table 2:** Selected processing parameters for the Master Je t2 spray gun.

Spray Parameters	Standard Flame	Reducing Flame	
	Coating (C1)	Coating (C2)	Coating (C3)
Acetylene pressure (bar)	1.4	1.4	1.4
Oxygen pressure (bar)	4	4	4
Gas flow (ball height: mm)	Acetylene: 84 Oxygen: 70	Acetylene: 99 Oxygen: 55	Acetylene: 99 Oxygen: 55
Air pressure (bar)	3–4	3–4	3–4
Spray distance (mm)	170	170	300
Spray angle	90	90	90
Feed speed	70 cm/min	70 cm/min	70 cm/min

**Table 3:** Corrosion tests result obtained in 3.5 wt. % NaCl solution.

Coatings	Ecorr (mV _SCE_)	Icorr (µA/cm^2^)	ßa	ßc
S	−318.098	84.66 ± 3.1	265.13 ± 4.1	122.16 ± 5.1
C1	−406.268 ± 4.2	5.844 ± 0.9	192.411 ± 3.8	132.406 ± 3.5
C2	−464.699 ± 6.7	3.865 ± 0.7	235.104 ± 3.3	132.506 ± 3.1
C3	−475.699 ± 5.1	2.027 ± 0.3	277.602 ± 4.9	132.603 ± 4.8

**Table 4:** Fitting results of EIS for coating C1 in 3.5 wt.% NaCl solution, simulated by the proposed equivalent circuit (EC).

Time	Rs (Ω·cm^−2^)	Rp (Ω·cm^−2^)	(CPE)c (Ω^−1^·cm^−2^s^−n^)	W (Ω^−1^·cm^−2^S^−0.5^)	(CPE)c (Ω^−1^·cm^−2^ s^−n^)	n	Rt (Ω·cm^−2^)	χ^2^ × 10^−4^
12 h	8.144 × 10^−3^	15.79	21.31	10.33	52.3	0.76	0.353	9.581
96 h	6.69	42.73	3.357 × 10^−2^	0.37	0.24	0.58	84.09	1.16

**Table 5:** Fitting results of EIS of coatings C2 and C3 in 3.5 wt. % NaCl solution, simulated by the proposed equivalent circuit (EC).

	Coating C2							
Time	Rs (Ω·cm^−2^)	Rpor (Ω·cm^−2^)	(CPE)c (Ω^−1^·cm^−2^·s^−n^)	(CPE)dl (Ω^−1^·cm^−2^·s^−n^)	n	Rt (Ω·cm^−2^)	W (Ω^−1^·cm^−2^·S^−0.5^)	χ^2^ × 10^−3^
12 h	9.662	14.44	0.16	0.2133	0.62	405	8.526	4.90
96 h	8.479	263.1	2.56 × 10^−3^	7.36 × 10^−3^	0.31	678.4	0.2285	6.79
	**Coating C3**							
**Time**	**Rs** **(Ω·cm^−2^)**	**Rpor** **(Ω·cm^−2^)**	**(CPE)c** **(Ω^−1^·cm^−2^·s^−n^)**	**(CPE)dl** **(Ω^−1^·cm^−2^·s^−n^)**	**n**	**Rt** **(Ω·cm^−2^)**	**W** **(Ω^−1^·cm^−2^·S^−0.5^)**	**χ^2^ × 10^−4^**
12 h	12.72	171.8	0.035	0.05	0.52	409.2	1.314	4.47
96 h	8.323	213.3	0.015	0.017	0.37	850.5	123.9	7.97

## Data Availability

Data are contained within the article.
